# P-612. Effectiveness of Live Attenuated and Inactivated Influenza Vaccines in Children: Data from the 2023/24 Influenza Season

**DOI:** 10.1093/ofid/ofae631.810

**Published:** 2025-01-29

**Authors:** Allyn R Bandell, Chris Barker, Oliver Dibben

**Affiliations:** BioPharmaceuticals Medical, AstraZeneca, Castle Rock, Colorado; AstraZeneca, Cambridge, England, United Kingdom; AstraZeneca, Cambridge, England, United Kingdom

## Abstract

**Background:**

Annual vaccination with a live attenuated influenza vaccine (LAIV) or inactivated influenza vaccine (IIV) is the most effective way to protect children from the burden of influenza infection, and to reduce the potential for transmission to family members and the community. Here we report vaccine effectiveness (VE) from data reported globally for LAIV and IIV in children from the 2023/24 influenza season.

**Figure:**
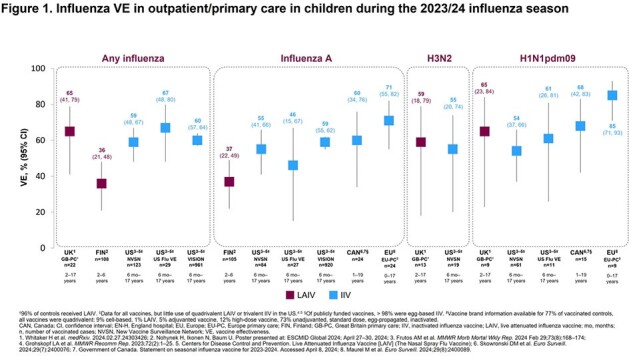

**Methods:**

Quadrivalent LAIV and IIV effectiveness studies conducted in children in the 2023/24 influenza season were identified from published literature and public health websites. Studies from Canada, Finland, the UK, the US, and a European-wide study in 10 countries, reporting VE data for any influenza infection (all strains), by strain type (all influenza A, A/H3N2, and A/H1N1pdm09), and by vaccination setting (primary/outpatient care and hospital) were included.

**Figure:**
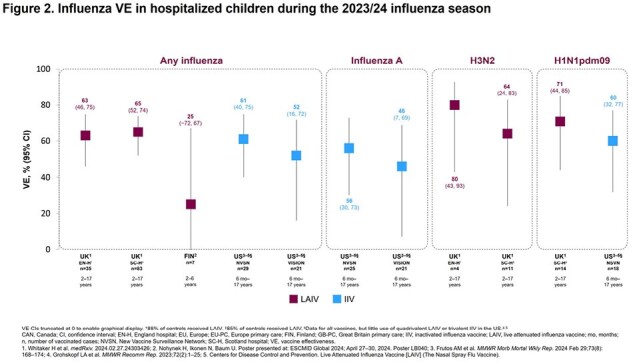

**Results:**

In five studies reporting on influenza infections (all strains) in children with ages ranging from 6 months to 19 years across studies, VE estimates for LAIV ranged from 36% (95% CI: 21–48) in Finland to 65% (95% CI: 41–79) in the UK, and for IIV ranged from 59% (95% CI: 48–67) to 67% (95% CI: 48–80) in the US. When analyzed by vaccination setting, VE against any influenza, influenza A, A/H3N2, and A/H1N1pdm09 in all the included countries ranged from 36–65% for LAIV and 46–85% for IIV in children in primary/outpatient care (**Figure 1**). For hospitalized children, VE against any influenza, influenza A, A/H3N2, and A/H1N1pdm09 in all the included countries ranged from 25–80% for LAIV and 46–61% for IIV (**Figure 2**). Where available, VE data against influenza A, A/H3N2, and A/H1N1pdm09 were comparable for both LAIV and IIV in children in primary/outpatient care and hospitalized children.

**Conclusion:**

VE data for the 2023/24 season from global reports show LAIV and IIV demonstrated comparable moderate protection for children against influenza infection. These early VE data were similar across the included healthcare settings of outpatient/primary care and hospitals.

**Disclosures:**

**Allyn R. Bandell, PharmD**, AstraZeneca: Stocks/Bonds (Public Company) **Chris Barker, PhD**, AstraZeneca: Stocks/Bonds (Public Company) **Oliver Dibben, PhD**, AstraZeneca: Stocks/Bonds (Public Company)

